# Protein family neighborhood analyzer—ProFaNA

**DOI:** 10.7717/peerj.15715

**Published:** 2023-07-21

**Authors:** Bartosz Baranowski, Krzysztof Pawłowski

**Affiliations:** 1 Department of Biochemistry and Microbiology, Warsaw University of Life Sciences, Warszawa, Poland; 2 Institute of Biochemistry and Biophysics, Polish Academy of Sciences, Warszawa, Poland; 3 Department of Molecular Biology, University of Texas Southwestern Medical Center, Dallas, Texas, United States; 4 Department of Translational Sciences, Lund University, Lund, Sweden

**Keywords:** Gene function prediction, Genomic neighborhoods, Protein domains, Comparative genomics

## Abstract

**Background:**

Functionally related genes are well known to be often grouped in close vicinity in the genomes, particularly in prokaryotes. Notwithstanding the diverse evolutionary mechanisms leading to this phenomenon, it can be used to predict functions of uncharacterized genes.

**Methods:**

Here, we provide a simple but robust statistical approach that leverages the vast amounts of genomic data available today. Considering a protein domain as a functional unit, one can explore other functional units (domains) that significantly often occur within the genomic neighborhoods of the queried domain. This analysis can be performed across different taxonomic levels. Provisions can also be made to correct for the uneven sampling of the taxonomic space by genomic sequencing projects that often focus on large numbers of very closely related strains, *e.g*., pathogenic ones. To this end, an optional procedure for averaging occurrences within subtaxa is available.

**Results:**

Several examples show this approach can provide useful functional predictions for uncharacterized gene families, and how to combine this information with other approaches. The method is made available as a web server at http://bioinfo.sggw.edu.pl/neighborhood_analysis.

## Introduction

### Gene neighborhood

The definition of the operon dates to 1960 when Jacob & Monod (1961) described the *lac* phenotype of *Escherichia coli*. The term operon refers to a genomic cluster of co-regulated genes in prokaryotes. They encode functionally related proteins that are under the control of a single regulatory signal or promoter. Numbers of highly conserved operons in genomes of bacteria or archaea are usually small, more often the operons are unique and rare (Rogozin et al., 2002; Gómez, Cases & Valencia, 2006; Osbourn & Field, 2009). The organization of genomes within bacterial and archaeal organisms is driven by the operonic principle. However, while the majority of prokaryotic gene clusters cannot be classified as operons due to local rearrangements, they often retain co-expression and co-functionality. Moreover, gene clustering into groups of similar or related functions also happens in eukaryotic genomes like yeast, plants, or mammals (Osbourn & Field, 2009; Szczepińska & Pawłowski, 2013; Slot & Gluck-Thaler, 2019; Razin et al., 2021). Some features of these groups of genes are comparable to prokaryotic operons, such as physical clustering and co-regulation. Despite differences in mechanisms of gene expression in eukaryotic and prokaryotic organisms, similar mechanisms may create selection pressure for the preservation of the sequential order of genes (Lawrence, 2002; Blumenthal, 2004; Michalak, 2008; Shafee & Lowe, 2017). Although the evolutionary mechanisms leading to the conserved clustering of genes in many, even distant, genomes are not completely understood, the likely causes include tandem duplication, shared chromatin domains, transcription read-through, and shared cis-acting elements. Evolutionary benefits include co-functionality, tissue specificity, and protein-protein interactions (Ohno, 1971; Batada, Urrutia & Hurst, 2007; Yi, Sze & Thon, 2007; Al-Shahrour et al., 2010; Lemay et al., 2012). As an example, locations of co-expressed genes in a genome are not random. It is well-known that genes involved in common biological functions tend to be also neighboring within genomes, *e.g*., members of protein complexes or elements of signaling or metabolic pathways (Lemay et al., 2012; Zaharia et al., 2019). Therefore, investigation of gene grouping within prokaryotic genomes can be a starting point to predict functions of uncharacterized genes, predict protein interactions, or analyze events by which genomes have evolved (Fong et al., 2008; Mihelčić, Šmuc & Supek, 2019). Despite widespread genomic clustering of functionally related genes, even more often genomic neighbors are unrelated functionally. Thus, a challenge is to develop methods to assess the statistical significance and biological relevance of genomic neighborhoods (Mihelčić, Šmuc & Supek, 2019). We present a novel tool for genomic neighborhood analysis that assesses which protein structural/functional domains are significantly often encoded in the genomic neighborhoods of the genes encoding the query domain. For brevity, we will be using the phrase “protein domains occurring in the genomic neighborhoods”. Currently, our database contains over 80,000 bacterial genomes. We analyze genomic neighborhoods in an unbiased manner, without predicting operon-like relationships, only focusing on physical genomic proximity.

### Existing bioinformatics tools for gene neighborhood analysis

Currently, there are many tools that offer a range of bioinformatics analyses of genomic neighborhoods built upon different databases. Here, we will briefly present a few software tools whereas one of the features is gene neighborhood analysis. COGNAT (Klimchuk et al., 2017) is a tool built on a database of manually curated representative sets of 711 completely sequenced prokaryotic genomes. For each genome, HMM profiles are used to map clusters of orthologous groups (COG) and Pfam domains onto proteins coded in the genome. The tool visualizes all genomes where the query COG or Pfam domain is found and presents all COG or Pfam domains that occur in the query neighborhoods if they satisfy selected occurrence thresholds.

The Gene Neighboring Scoring Tool (G-NEST) (Lemay et al., 2012) is a standalone software. Pre-made G-NEST database contains syntenic blocks for ten mammalian genomes, but users can upload their own syntenic blocks for genomes of interest. As input, the user provides gene expression data and genomic locations for query genes. Then a pipeline builds all possible gene neighborhoods in a user-defined range of neighborhood sizes. Gene expression data is used to calculate the correlation of expression for every query gene and every other gene in the genome. Results from these two analyses are then used to rank putative neighborhoods in terms of the neighborhood synteny conservation and correlated expression within the neighborhood.

Probably the most advanced and broadly used tool for gene neighborhood analysis is STRING. In its current version (11.5), the database contains data about 14,000 genomes of which nearly 90% are prokaryotic. The STRING neighborhood analysis tool searches for homologs of the query gene(s) in all genomes. The software predicts functional partners based on combined scores from conserved presence in genomic neighborhoods, gene fusion, co-occurrence in genomes, and co-expression, as well as information from functional databases and text mining (Szklarczyk et al., 2019).

The next interesting tool for the analysis of gene neighborhoods is FlaGs. It compares conservation of gene neighborhoods across evolution. For an input set of proteins, it clusters sequences found in their genomic neighborhoods using the sensitive sequence search algorithm, Jackhmmer. For ease of interpretation, it provides a phylogenetic tree of the input proteins annotated by the conserved genomic neighbor clusters (Saha et al., 2021).

Another tool, SLING, can be used to search for divergent occurrences of functionally related genomic neighborhoods by requiring the presence of a conserved “hit” gene together with predefined “partner” (neighbor) genes (Horesh et al., 2018).

The next three tools take a different approach to neighborhood analysis because they focus on visualization. The Microbes Online portal provides information about over 1,700 prokaryotic genomes. It integrates functional genomic data and comparative genomic analysis providing a structured view for comparative genomics (Dehal et al., 2009). This site allows the analysis of gene neighborhoods based on the physical position of the query gene on the chromosome across distant species. Results can be presented in two types of tree-based views: the first one shows genes neighborhood conservation and groups neighborhoods by the presence of different domains; the second view shows taxonomy groups in which the query gene was found.

Integrated Microbial Genomes & Microbiomes (IMG/M) is a database that makes available information about over 80,000 prokaryotic genomes. This portal does not provide neighborhood analysis, however, it allows to visualize genomic neighborhoods of homologs of query genes with functional annotations (*e.g*., coloring by COG or Pfam domains) (Chen et al., 2019). BioCyc is a database collection including 19494 pathway and genome databases from Archaea, Bacteria, and Eukarya. Despite a large set of tools and the size of the whole database, BioCyc does not have a ready tool or pipeline for direct statistical neighborhood analysis; however, it offers comparative analysis by visualization of selected datasets, which can be the genomic context of selected genes and its homologs (Karp et al., 2019).

EFI-GNT is a tool that uses the sequence similarity network (SSN) as an input. SSN is generated by other EFI tools available on their site. This network is created based on found homologs of query protein sequence in bacterial, archaeal or fungal organisms. Similarities and differences from alignments are used to calculate edges between homologs and related proteins are grouped together in clusters. Then, for each sequence in SSN, the algorithm collects domains from the closest neighborhood. Returned data is used to create networks that show the presence of Pfam domains in each SSN and its reversed visualization where is shown in which SSN each domain is present (Zallot, Oberg & Gerlt, 2019).

Currently, the amount of biological data produced thanks to advances in high-throughput technologies is huge. Easy access to data creates opportunities to develop new analysis tools. However, for a productive insight, not only visualization but also appropriate statistical evaluation is necessary (Lee, 2010). Of all the tools mentioned above, only EFI-GNT, and to some extent STRING, provides proper statistical analysis.

In this article, first, we present the statistical approach for functional analysis of gene neighborhoods. Then, we offer an overview of how much information this approach may provide for the thousands of proteins containing “domains of unknown function” (DUFs). Next, we discuss in more detail functional predictions for several such DUFs, one of which has been recently experimentally characterized. Finally, we compare our method with the popular STRING server.

## Materials and Methods

### Availability and code

The algorithm, instructions and sample results of the presented tool are available at http://bioinfo.sggw.edu.pl/neighborhood_analysis. Code of ProFaNA algorithm without data is stored at https://www.github.com/steven0seagal/ProFaNA (DOI 10.5281/zenodo.7993764).

### Source of data

All bacterial genome sequences available from IMG/M—Integrated Microbial Genomes and Microbiomes service (https://img.jgi.doe.gov/) were downloaded in December 2020. In this database, genomic information on archaea, bacteria, eukaryotes, plasmids, and viruses are stored. The current IMG/M database (as of December 2020) contains 88,754 bacterial genomes (Chen et al., 2021). In our internal database, we use all genomes available in IMG. Due to the constant growth of the IMG/M database, we are planning to update our genome collection regularly. Additionally, we have incorporated the functionality to map genomes into the NCBI RefSeq database. Thus, ProFaNA analysis can be performed on “all IMG genomes” or “reference genomes” (from NCBI RefSeq). In IMG, all genes from every genome were annotated based on the presence of Pfam domains in the proteins encoded by each gene. Also, each gene was annotated by the genomic contig in which it was found (Chen et al., 2021). In our approach, we decided to use the Pfam database and protein family classification as the elementary object in the neighborhood analysis. As of February 2022, the number of nucleotide sequences in GenBank is over 236 million, and over 1.75 billion in Whole Genome Sequencing projects in NCBI (Sayers et al., 2019). It is impossible to characterize all their functions experimentally. Here, a standardized functional domain classification, like Pfam, can be of help. The Pfam classification, developed for over 20 years, provides protein domain definitions and functional descriptions. Matching sequences to Pfam entries allow transferring information about function from experimentally characterized proteins to uncharacterized ones. The current version of the Pfam database (March 2021) includes 19,179 protein families (El-Gebali et al., 2019). Thus, using Pfam classification to describe gene function allows us to functionally annotate the whole genome in a concise and structured way. Pfam descriptions combined with statistical methods enable analyses of protein domain co-occurrence in genomic neighborhoods which is the focus of this tool.

### Matching Pfam to Gene Ontology

The short descriptions of Pfam domains delivered by ProFaNA are not sufficient for understanding function. Thus, predicting the function of the query domain requires additional database and literature mining. To help users interpret results, the output of ProFaNA is annotated by linking significantly overrepresented Pfam domains to Gene Ontology (GO) annotations, using mapping data from GeneOntology.org (Ashburner et al., 2000; Carbon et al., 2021). The GO information is provided in three dimensions of the classification: molecular function, cellular component, and biological process. Thus, GO term identifiers, term descriptions, and related Pfam domains are provided for all the overrepresented Pfam domains. The provided matching may be helpful as a starting point for further investigation notwithstanding the inherent difficulty of assigning function to individual domains.

### Analysis scenarios and input data

**Figure 1:**
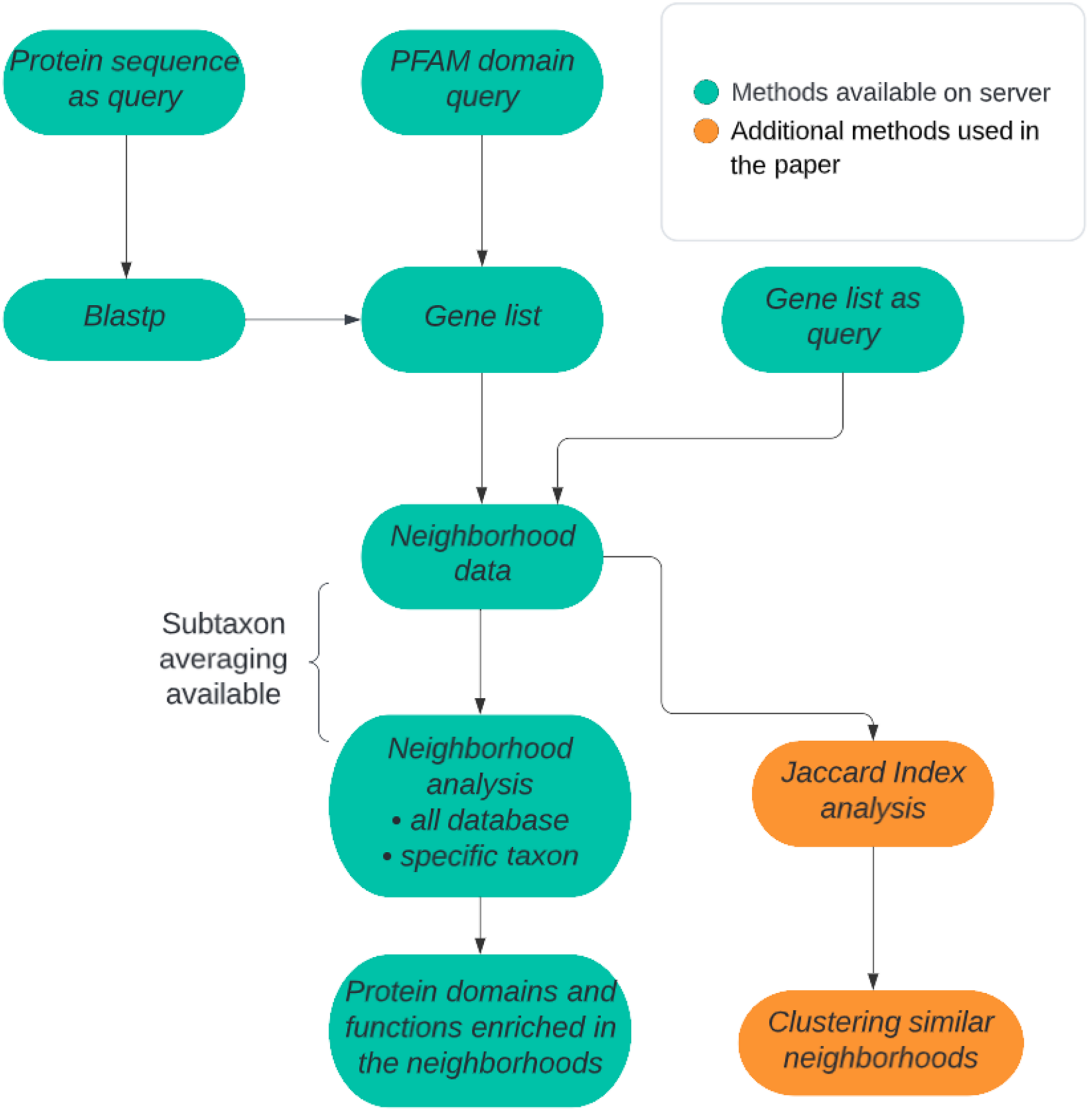
ProFaNA flowchart. The green boxes correspond to analysis steps implemented on the ProFaNA server, and the orange boxes indicate additional analyses used in the article.

There are two alternative approaches available in our method: (1) prokaryotic all-genomes neighborhood analysis or (2) prokaryotic taxon-specific neighborhood analysis. In the case of the first approach, either all genomes may be treated equally, or averaging can be performed at selected taxonomic levels: genus, family, order, class, or phylum (Fig. 1). The query can be a Pfam domain (whereas by applying IMG mapping it is translated into a list of all the genes coding for proteins containing the query domain), a user-defined list of genes or a protein sequence for which homologous sequences are found using Blastp algorithm from the DIAMOND tool (Buchfink, Xie & Huson, 2015). In the second approach, the genes need not be homologs. Typically, a gene list will represent proteins possessing a novel domain, not yet included in the Pfam domain database, and obtained from a sequence search, *e.g*., BLAST or HHblits. Either way, the query is always translated into a set of genomic neighborhoods. The necessary input information includes, besides the queried domain or gene list, the size of the genomic neighborhood to be investigated described as the number of base pairs around the query gene. Typically, the size of a neighborhood set to 5,000 bp in each direction (10 kbp of genomic sequence) corresponds typically to approx. A total of 10 genes in the whole neighborhood. Although we expect most operon-like functional units will not exceed 10 genes, this parameter can be changed by the user. In taxon-restricted analysis, taxa available for neighborhood analysis (genera or families) were chosen by requiring a large number of available genomes. Due to the possibility of a high false discovery rate that can be a serious problem when multiple tests are performed (*e.g*., many Pfam domains are evaluated for overrepresentation), the user can decide to apply a multiple test correction such as Bonferroni or Benjamini-Hochberg correction (Thissen, Steinberg & Kuang, 2002; Sedgwick, 2012).

### Statistical evaluation of the significance of occurrences of a “target” domain in the query neighborhoods

**Figure 2:**
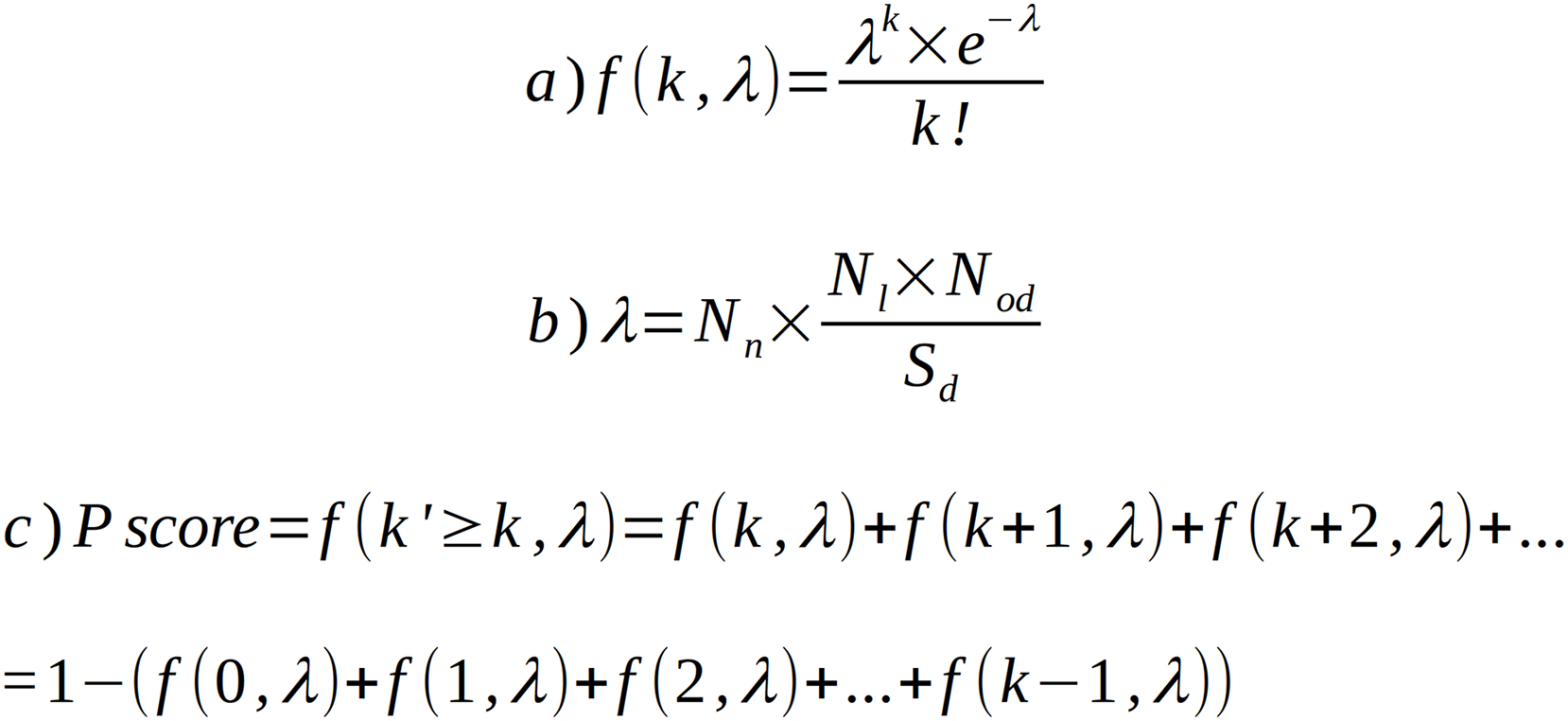
Poisson distribution equation and its modifications used to calculate domain occurrence probability score (*P* score). (A) Poisson distribution equation (probability mass function) for observing k occurrences of target domain D in query neighborhoods if the expected number of occurrences equals λ. (B) Method used to calculate λ in our algorithm, Nn—number of query domain neighborhoods, Nl—neighborhood length, Nod—number of occurrences of target domain in database, Sd—size of database. (C) Extension of equation (a) to calculate *P* score for k or more occurrences. Nl and Sd are expressed as numbers of genes.

The ProFaNA tool collects information about protein domain occurrence in prokaryotic neighborhoods of the query genes and, overall, in all the genomes. For each target domain from each query genomic neighborhood, we ask what the probability is of observing *k* or more target domain occurrences in *n* query neighborhoods by chance, *i.e*., if the target domain was randomly distributed in the genomes. To this end, we calculate the probability mass function using a modified Poisson distribution (Fig. 2A). Here, the expected number of events (λ) is the product of the number of analyzed query neighborhoods (N_od_) and the probability of finding the target domain in a randomly selected genomic region of the size of a neighborhood. *k* is the observed number of occurrences of the target domain in query domain neighborhoods (Fig. 2B). We decided to exclude domains that occur in neighborhoods less often than in a random neighborhood. The sizes of neighborhoods and genomes are expressed as numbers of genes.

To obtain the probability (*P* score) of observing *k* or more occurrences of the target domain by chance, we subtract from unity the sum of probabilities for cases where the number of occurrences *k’* is less than *k* (Fig. 2C). The output is a tabular text file including target domain IDs, names and brief descriptions, the *P* score of the target domains, numbers of target domain occurrences in query neighborhoods, and numbers of target domain occurrences in all analyzed genomes. The “neighborhood percentage” column informs, in what fraction of the query neighborhoods the target domain is found. Sub-taxon averaging is intended to address uneven genome sampling. The uneven sampling of microbial strains within different taxonomic groups by genome sequencing may have a very profound effect on genomic neighborhood analysis. Here, to minimize the adverse effects of this problem, we implemented an optional averaging solution.

In our method, during the analysis of a higher-level taxonomy group (*e.g*., class) that consists of many lower-level sub taxa (*e.g*., orders) we conduct neighborhood analysis and collect results within sub taxa (*e.g*., orders) to extract values needed to calculate Poisson distribution for each target domain. Then, we identify the subtaxon with the largest number of query neighborhoods (n-sub_max_). For all the other subtaxa “i”, we calculate the difference between n-sub_max_ and n-sub_i_. Then, to assign equal weight to every subtaxon, we randomly draw (n-sub_max_−n-sub_i_) neighborhoods from subtaxon “i” and include them in the analysis. This modification is available as an option in two types of analysis: for taxon-specific analysis—at the family level (averaging over genera is always applied) and at the all-database level where the user can select whether an averaging method will be used or not. At the family level, 12 families are available, each containing more than 1,000 genomes. In the “all database” analysis, averaging can be performed on five taxonomy levels (genus, family, class, order, phylum). At each level, subtaxa containing at least 25 genomes are considered for the averaging analysis. For each of these taxonomy levels, we perform neighborhood analysis for every taxon (*e.g*., averaging on class level means that neighborhood analysis will be performed 86 times because that many subtaxa (classes) that include more than 25 genomes are present in the database).

### Additional neighborhood similarity analysis

**Figure 3:**
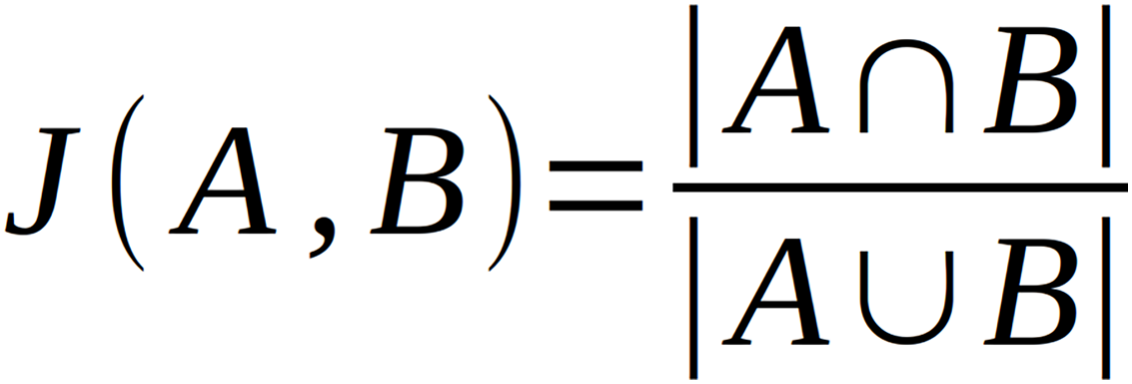
Equation for calculating Jaccard Index. A and B represent the sets of data to be compared.

The main analysis pipeline described above treats domains identified in the neighborhoods separately and does not consider neighborhood compositions. To analyze neighborhood composition and similarities, we extended our neighborhood analysis by applying Jaccard index-based clustering method (Kriegel, Kröger & Zimek, 2009). Having a set of neighborhoods of a query domain, one can calculate Jaccard Index for every neighborhood against every neighborhood. This statistical method is used to compare sets by measuring the similarity of their composition as the ratio of the intersection of two sets and the sum of these sets (Fig. 3).

The values of this statistic are between 0 and 1 where 0 means that compared sets are completely different and 1 means sets are identical (Real & Vargas, 1996). For a set of neighborhoods, the values of the Jaccard Index can be placed in a neighborhood similarity matrix which can be clustered.

To group similar neighborhoods, we used the agglomerative hierarchical clustering method. To obtain the distance between clusters of genomic neighborhoods, we calculated the pairwise distance between sets of observations using the Single linkage clustering (also called the Farthest Point Algorithm or Voor Hees Algorithm) (Defays, 1977; Nielsen, 2016; Virtanen et al., 2020). Thus, clusters of neighborhoods that are similar in protein domain composition tend to group together. The clusters can also be annotated by taxonomy information to get insight into neighborhood conservation across different groups of organisms.

### ProFaNA—comparison with STRING

The main reason to create the ProFaNA tool was to develop an algorithm that will help analyze prokaryotic genomic neighborhoods to guide experiments. For comparison of ProFaNA with STRING, we focused on domains of unknown function (DUFs) present in *Escherichia coli* K-12. For each query DUF, we use ProFaNA to identify a list of domains significantly often occurring in its neighborhoods. Then, using the STRING database, we identify *E. coli* proteins that contain the queried domain and collect domains that are identified by the STRING algorithm as functionally related. In the end, we compare the lists of related domains identified by STRING and by ProFaNA. This approach was used for a global comparison of the most abundant DUFs.

### Site & user interface

The ProFaNA server discussed in this article can be accessed through its dedicated website, which can be found at http://bioinfo.sggw.edu.pl/neighborhood_analysis/. Upon reaching the website, users are given the option to choose between three input formats: Pfam domain ID, list of genes, or protein sequence. Depending on the chosen input format, different forms containing pre-defined parameters open up to facilitate analysis.

If the user selects the gene list as their query input, they must provide the server with a list of genes (IMG identifiers), the size of the neighborhood to be analyzed, the multiple test correction method for statistics, a choice of DNA strand for analysis, the cut-off for *P* scores, and an analysis name. If the user chooses the Pfam domain ID as their query input, they must provide the Pfam domain identifier and select a group of organisms to be searched for the presence of the queried domain. The rest of the input fields are the same as those for the gene list option. In addition to the Pfam domain ID and gene list input options, the ProFaNA server also allows users to input protein sequences in FASTA format to search for homologs using a DIAMOND search. Additionally, the E-value threshold for sequence similarity has to be selected. The rest of the fields are the same as those for the gene list option.

Once the analysis is complete, users can expect to receive an email notification with a link to their results. This email notification system helps users stay informed about the progress of their analysis, especially when the calculation time is longer than expected. After receiving the email notification, users can access their results on the ProFaNA website. The results are displayed in a user-friendly table that includes information about target domain Pfam IDs, domain names and brief descriptions, *P* scores, and the numbers of occurrences in query neighborhoods and in all analyzed genomes. The “percentage in neighborhoods” column provides information about the fraction of query neighborhoods in which the target domain is found. Information about Gene Ontology (GO) terms for each target domain is also included, if available.

Users can easily search through all values in the output table, and the table can also be downloaded in CSV format for further analysis. Additionally, we provide a taxonomy report that shows in which genes and genomes the query domain was found. This can be useful for users that want to make additional analysis on a smaller taxonomy group of choice.

### Updates

We are aware that IMG Pfam annotation is not up to date, and it is a downside of our tool, however, as soon as IMG/JGI updates its database with up-to-date Pfam domain annotations, we will update our records as well. The process of updating our software database with data from IMG/JGI website involves multiple steps. Firstly, existing and new data are compared and any discrepancies are identified. Next, new data is downloaded and integrated into the ProFaNA database while any deprecated data is removed. Statistics are then prepared for the updated database. The entire process can take up to a week, depending on the size of the database and the amount of new data.

## Results

### Overview of ProFaNA results for uncharacterized protein domains

**Figure 4:**
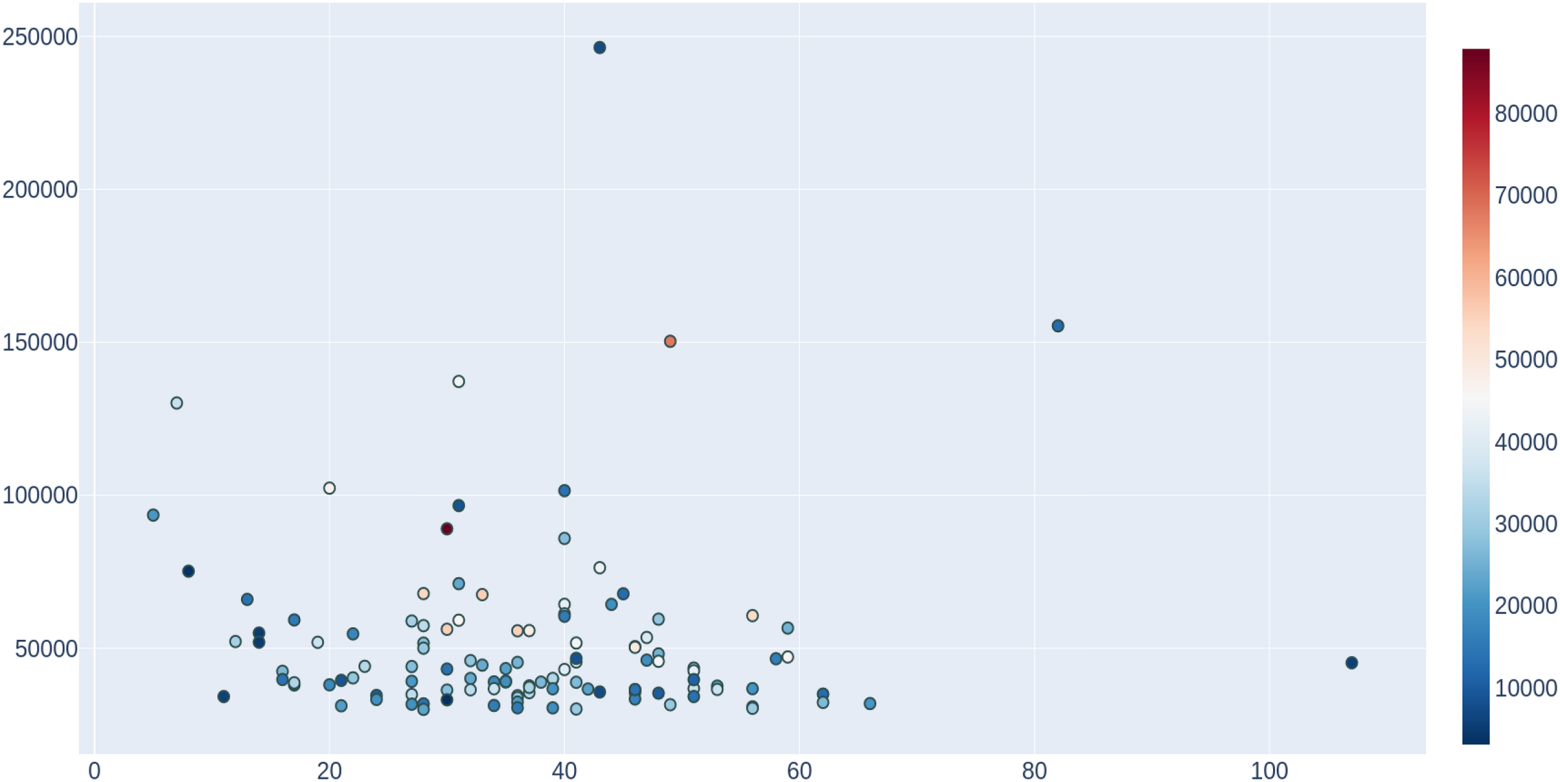
Analysis of domains of unknown function (DUFs) in the ProFaNA database. For the 100 DUFs with most occurrences in the IMG database, significant neighborhood domains were counted (with a *P* score value lower than 0.00001 and neighborhood presence higher than 5%). The number of significant domains in the neighborhood (x-axis) plotted *vs*. the number of occurrences of the query DUF in the whole database (y-axis) and the number of genomes in which the domain was identified (color bar).

To provide an estimate of the potential usefulness of ProFaNA, we performed an “all-database” neighborhood analysis for 4,012 DUFs from the Pfam database that are present in more than 10 genomes. For the query DUF domains, ProFaNA provided between 13 and 370 domains significantly overrepresented in their neighborhoods (*P* score below 10^−5^ and present in at least 5% of query neighborhoods). Typically, these significant domains occurred in between five and 60% of neighborhoods. Additionally, from the previous analysis we considered the 100 most common DUF domains in the database and considered the distribution of significant domains (Fig. 4). In a plot of query domain occurrences *vs*. the number of significant neighborhood domains, there is no dominant trend. Potentially the most interesting DUF domains are located on the left side of the plot *i.e*., a number of significant neighborhood domains lower than 30 for “common” query domains occurring in the database between 50,000–100,000 times. In contrast, the right side of the plot contains query domains with vast numbers of significant neighborhood domains with a relatively small number of occurrences in the database and a number of genomes, which can mean many different neighborhoods in different organisms.

To assess results on a smaller dataset, we used all Pfam domains that are present in any *Enterobacteriaceae* genome as a query, but excluding the *Escherichia* genus to minimize impact of high number of *Escherichia coli* genomes on this analysis. The numbers of significant neighborhood domains that occur more than once in query neighborhoods ranged between two and 145, these domains typically occurred in more than 5% of all analyzed neighborhoods.

To assess ProFaNA performance in individual cases, we performed the analysis for one protein domain with a recently characterized function and two uncharacterized domains for which functions were predicted by protein structure prediction and homology detection.

### SelO

A prototype of the ProFaNA approach was used to study the predicted pseudokinase, selenoprotein O (SelO) (Dudkiewicz et al., 2012). It was predicted that SelO adopts a kinase 3D structure, and is involved in oxidative stress response (Dudkiewicz et al., 2012). SelO is one of the protein kinase superfamily’s most conserved member (Galperin & Koonin, 2004; Dudkiewicz et al., 2012; Sreelatha et al., 2018). In the prototype analysis of genomic neighborhoods of bacterial SelO homologs, overrepresented genes were found to be involved in ABC transport system, basic metabolism regulation and oxidative stress response (Dudkiewicz et al., 2012).

Subsequent experimental studies confirmed most of these predictions. The crystal structure of SelO indeed showed the predicted protein kinase-like fold. Further, it was shown that unlike kinases, SelO transfers AMP instead of phosphate to protein substrates and SelO-mediated AMPylation is involved in redox homeostasis and oxidative stress response (Sreelatha et al., 2018).

Here, the SelO pseudokinase domain was used as query in ProFaNA neighborhood analysis to find domains that occur in its vicinity, in genomes within the genera *Escherichia, Salmonella, Shigella, Klebsiella*, within the family *Enterobacteriaceae* and in the whole database (order, class, family, genus and phylum averaging, and also without averaging) (Data S1, S2, S6–S13 and S23).

Analyzing the ubiquitous SelO homologs in all *Escherichia* genomes, we found 17 domains significantly overrepresented in the neighborhoods. Sixteen of them occur in SelO neighborhoods in more than 85% of *Escherichia* genomes. Thus, they form a conserved SelO neighborhood typical for the *Escherichia* genus and may be related functionally to SelO. Additionally, in this group, eight domains do not occur in the analyzed genomes outside SelO neighborhoods (Data S1, Figs. S1 and S2). These domains include glutathione peroxidase domain; tRNA synthetase B5 domain; kinase/pyrophosphorylase. Functionally, this set of domains suggests that the SelO domain could be related to oxidative stress response and translation. In terms of molecular functions from Gene Ontology (GO) classification, significant domains are matched to 10 different GO IDs, including phosphate cycles in cell, DNA/RNA processes, oxidative stress response, magnesium ion binding and transmembrane transport. Accordingly, clustering analysis of these neighborhoods showed that 90% of neighborhoods form a single, conserved group (Fig. S3).

Neighborhood analysis of the SelO domain within genomes from the *Shigella*, *Salmonella* and *Klebsiella* genera yielded results very similar to *Escherichia*.

**Figure 5:**
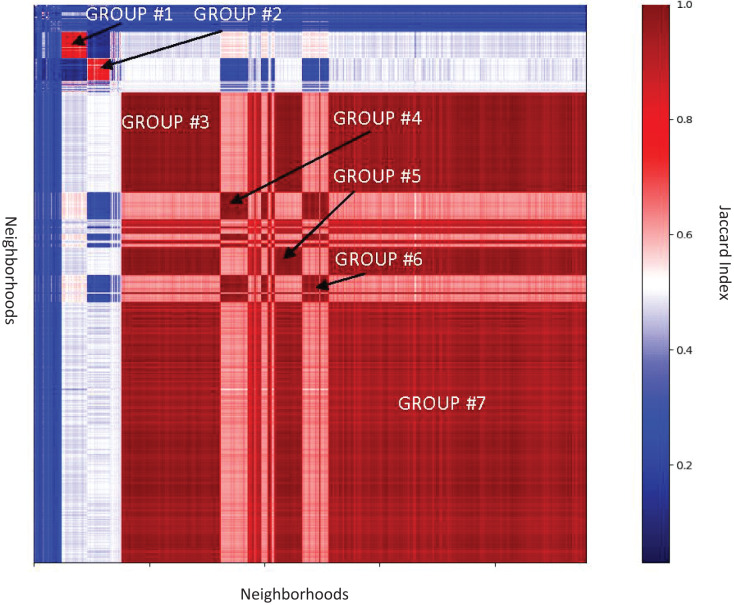
Similarities between genomic neighborhoods for the SelO domain in Enterobacteriacae genomes. Total of 10,773 neighborhoods shown. Rows and columns correspond to the neighborhoods, color bar shows value of Jaccard Index, representing similarity. The heatmap is clustered using single linkage clustering.

In neighborhood analysis of the *Enterobacteriaceae* family there is only one domain not found in the individual genera analysis, Phage_integrase, while nine domains from genera analysis were not found in family analysis (Data S2). Clustering of SelO neighborhoods in *Enterobacteriaceae* split them into seven highly similar groups (Data S14–S20, Fig. 5), typically specific for genera.

Additionally, we conducted neighborhood analysis of the SelO domain using the all-genomes neighborhood analysis, averaging on order level (Data S9). Thus, ProFaNA identified 4,848 overrepresented domains in 45,479 SelO neighborhoods. Five domains that occur in most of neighborhoods, HATPase (34%) HisKA (30%) are found in histidine kinases, ABC_trans (33%) is one the most abundant domains in bacteria and is found in transmembrane transporters, Response_reg domain (34%) found in two-component systems and Pyr_redox 2 (26%) domain of peroxidases. The same analysis was performed with averaging on class, family, phylum, and genus level with similar results (Data S10–S13). Considering all these functions, the SelO domain may be involved in regulating cellular transport, oxidative stress response and response to other stimuli.

Clustering of SelO neighborhoods from all genomes available in our database shows two very consistent groups (Fig. S3). The first one contains 8,260 neighborhoods where three main genera are *Escherichia* (44%), *Salmonella* (36%) and *Klebsiella* (14%). In this group, there are six domains that are found only in the proximity of SelO domain: hemP, GSHPx, B5, PPDK_N, Kinase-PPPase, FDX-ACB (Data S21). The second group contains 1,550 neighborhoods, almost all from the *Acinetobacter* genus. For this group, the unique domain is Cob_adeno_trans, which is found in cobalamin adenosyltransferases that convert inactive cobalamins to adenosylcobalamin (Data S22).

In the SelO neighborhood analyses presented above, regardless of the taxonomic levels used, we can notice that some domains occur only once per genome, and they are in a close vicinity of the query domain (SelO/YdiU). These domains form a conserved co-localized group. First, two kinase domains PPDK_N and Kinase-PPPase suggest that the SelO domain function may be related to phosphorylation. The other two domains are GSHPx found in glutathione peroxidase, and hemP responsible for uptake of hemin as source of iron. Last three domains, B3_4, B5, FDX-ACB are found in Phe-tRNA synthetase and it was proven that oxidation of Phe-tRNA synthetase positively regulates mechanisms that control translation process and help cell to properly respond to oxidative stress (Steiner, Kyle & Ibba, 2019). To sum up, we can speculate that the SelO domain is located in genomic regions where genes are translated in response to oxidative stress.

### DUF5565

This uncharacterized domain, evolutionarily conserved in animals, is also found in some bacterial genomes. To conduct neighborhood analysis, bacterial homologs were collected from the IMG/JGI database using BLAST search. Thus, 390 homologs were used as input for ProFaNA neighborhood analysis. Among the protein domains significantly overrepresented (*P* score < 0.00001) in the DUF5565 neighborhoods, 10 were found in at least 5% of neighborhoods.

Of those 10 domains, seven are either -nucleic acid binding or have RNase, RNA ligase, or RNA polymerase function, which suggests a functional link between RNA and the DUF5565 domain. Having made a serendipitous observation that several tRNA genes occur in some DUF5565 neighborhoods, we decided to test the significance of this observation, although ProFaNA normally does not consider genes that do not encode proteins. Indeed, tRNAs genes were found to be significantly overrepresented (*P* score < 0.00001) in DUF5565 neighborhoods (Data S24). In fact, tRNA genes were found in more than 20% of DUF5565 neighborhoods which supports our functional prediction based on protein domains in the neighborhoods and additionally points at a tRNA-related function. In addition, among Gene Ontology terms returned for the significant domains, “RNA binding” supports the prediction. In fact, a recent preprint reports experimental data showing that C12ORF29, the human protein containing DUF5565, is a RNA ligase (Yuan et al., 2023).

### DUF4174

For this uncharacterized domain, found in vertebrates and numerous bacteria, oxidoreductase function was predicted by structural bioinformatics (Pawłowski et al., 2010). A neighborhood analysis of DUF4174 (PF13778) in the whole database returns 25 domains significantly overrepresented in neighborhoods and found in more than 10% of the neighborhoods.

Results suggest that functionally DUF4174 can be related to a few groups, histidine kinase, nitrite/sulfite metabolism, membrane transportation, DNA polymerase, and biotin carboxylase (Data S25). Out of 25 significant domains, 21 are found in the Gene Ontology classification molecular level. They can be linked into four groups: binding capability of different molecules, enzymatic activity (including for example oxidoreductase activity or DNA-binding transcription factor activity), transmembrane transporter activity and kinase activity.

Additionally, we analyzed the DUF4174 neighborhoods in genomes of the well-known pathogen *Mycobacterium* sp. Here, ProFaNA yielded six significantly overrepresented neighborhood domains in 22 neighborhoods. Despite a low number of neighborhoods and returned domains, one domain occurs in 95% of analyzed neighborhoods, CIA30, whose function was characterized in *H. sapiens* as an assembly factor for mitochondrial complex, supporting DUF4174 oxidoreductase role in these bacteria, possibly in a larger complex mediated by CIA30.

### Comparison with STRING database

To compare ProFaNA with established methods for genomic neighborhood analysis, we decided to use the popular STRING service which combines neighborhood information with other lines of evidence to predict functional relationships between genes.

**Figure 6:**
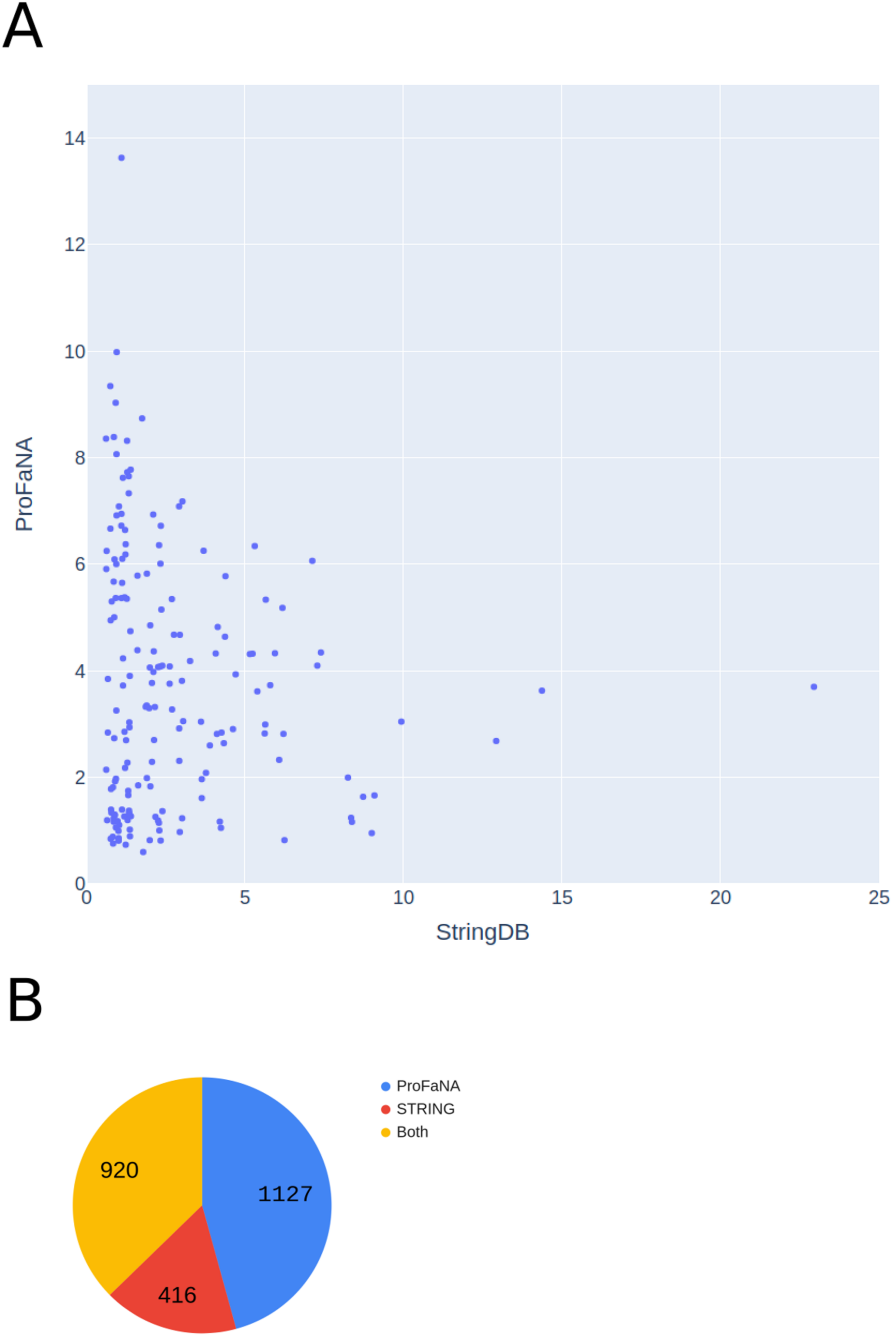
Comparison of numbers of unique functional relations provided by ProFaNA and StringDB. Comparison of numbers of unique functional relations between genes provided by ProFaNA and StringDB for all protein domains found in *Escherichia coli* str. K-12 MG1665. (A) The axes represent the numbers of unique relations obtained using ProFaNA (y-axis) and STRING database (x axis). (B) Numbers of functional relations obtained uniquely from ProFaNA analysis, uniquely from STRING database, and from both approaches. Total number of ProFaNa results = “ProFaNa” + “Both”.

First, we considered all Pfam domains present in the best-known *Escherichia coli* strain, K-12 MG1665, and analyzed their neighborhoods with ProFaNA. Thus, we analyzed over 4,000 domains from nearly 1,900 genes. For each protein domain, we recover domains that are significantly overrepresented (as per *P* score and query neighborhood presence rate). Then we mapped significant domains to genes and created a gene relationship network. Analogous gene-gene relations were downloaded from the STRING database. Comparing the two networks, a substantial number of relations (920, or 37%) are captured by both approaches (Fig. 6A). However, ProFaNA discovers uniquely 1,127 relations (almost half of the total number). On average, the ProFaNA algorithm returns 1.19 unique relations per gene, while the STRING database—only 0.44, which is a statistically significant difference according to the paired t-test (Fig. 6B). Differences between STRING and ProFaNA results can stem from several reasons: different definitions of genomic neighborhoods (STRING has a more conservative definition, requiring very short distance between adjacent genes), also the two tools differ in the sets of *E. coli* genomes considered and in the details of statistical evaluation of significance of co-occurrence in neighborhoods.

## Conclusions

The ProFaNA tool is, to our knowledge, the only one that allows analyzing prokaryotic genomic neighborhoods on a large scale, applying statistical evaluation, including thousands of genomes. Our organism dataset was made based on the IMG/JGI Portal, currently one of the biggest public microbial genomes databases. Our algorithm can be used to analyze the genomic neighborhoods of protein domains for genera or at higher taxonomic levels, while considering uneven distribution of sequenced organisms by application of an optional averaging procedure. Comparison with the STRING database suggests that ProFaNA can broaden the functional relationship networks. One limitation of the current version is its reliance solely on Pfam domains to characterize neighboring proteins. This tool is designed for scientists who want to explore the genomic neighborhood of a query domain, search for patterns of domain genomic proximities in different taxonomy groups or try to predict domain function based on neighborhood co-occurrence with better-characterized domains.

## Supplemental Information

10.7717/peerj.15715/supp-1 Supplemental Information 1 Neighborhood analysis of SelO domain. Comparison of significant domains found in genus-level and family-level analysis. Green circle represents significant domains from results for *Escherichia* sp., *Salmonella* sp. and *Klebsiella* sp. genomes, red circle represents domains from the *Enterobacteriaceae* family.

10.7717/peerj.15715/supp-2 Supplemental Information 2 Similarities between genomic neighborhoods of the SelO domain in the all genomes analysis. Total of 42691 neighborhoods shown. Rows and columns correspond to the SelO neighborhoods, color bar shows value of Jaccard Index, representing similarity in protein domain composition of the neighborhoods. The heatmap is clustered using single linkage clustering.

10.7717/peerj.15715/supp-3 Supplemental Information 3 Similarities between genomic neighborhoods of the SelO domain in analysis of genomes of the Escherichia genus. Total of 4130 neighborhoods shown. Rows and columns correspond to the SelO neighborhoods, color bar shows value of Jaccard Index, representing similarity in protein domain composition of the neighborhoods. The heatmap is clustered using single linkage clustering.

10.7717/peerj.15715/supp-4 Supplemental Information 4 Clusters of SelO neighborhoods in the *Enterobacteriaceae* family. Related to Figure 5. Featured domains are those that occur in genomes in close proximity of SelO but very rarely elsewhere in the genomes.

10.7717/peerj.15715/supp-5 Supplemental Information 5 Additional result tables.

## Additional Information and Declarations

### Data Availability

The following information was supplied regarding data availability: The code is available at GitHub and Zenodo:
https://github.com/steven0seagal/ProFaNA
steven0seagal. (2023). steven0seagal/ProFaNA: ProFaNA v.1.0 (1.0). Zenodo. https://doi.org/10.5281/zenodo.7993764
