# Supplementary figures and images for: Protein family neighborhood analyzer—ProFaNA
Source: PeerJ. 2023 Jul 21;11:e15715. doi: 10.7717/peerj.15715 (PMC10364804; doi:10.7717/peerj.15715)

Domain occurrence

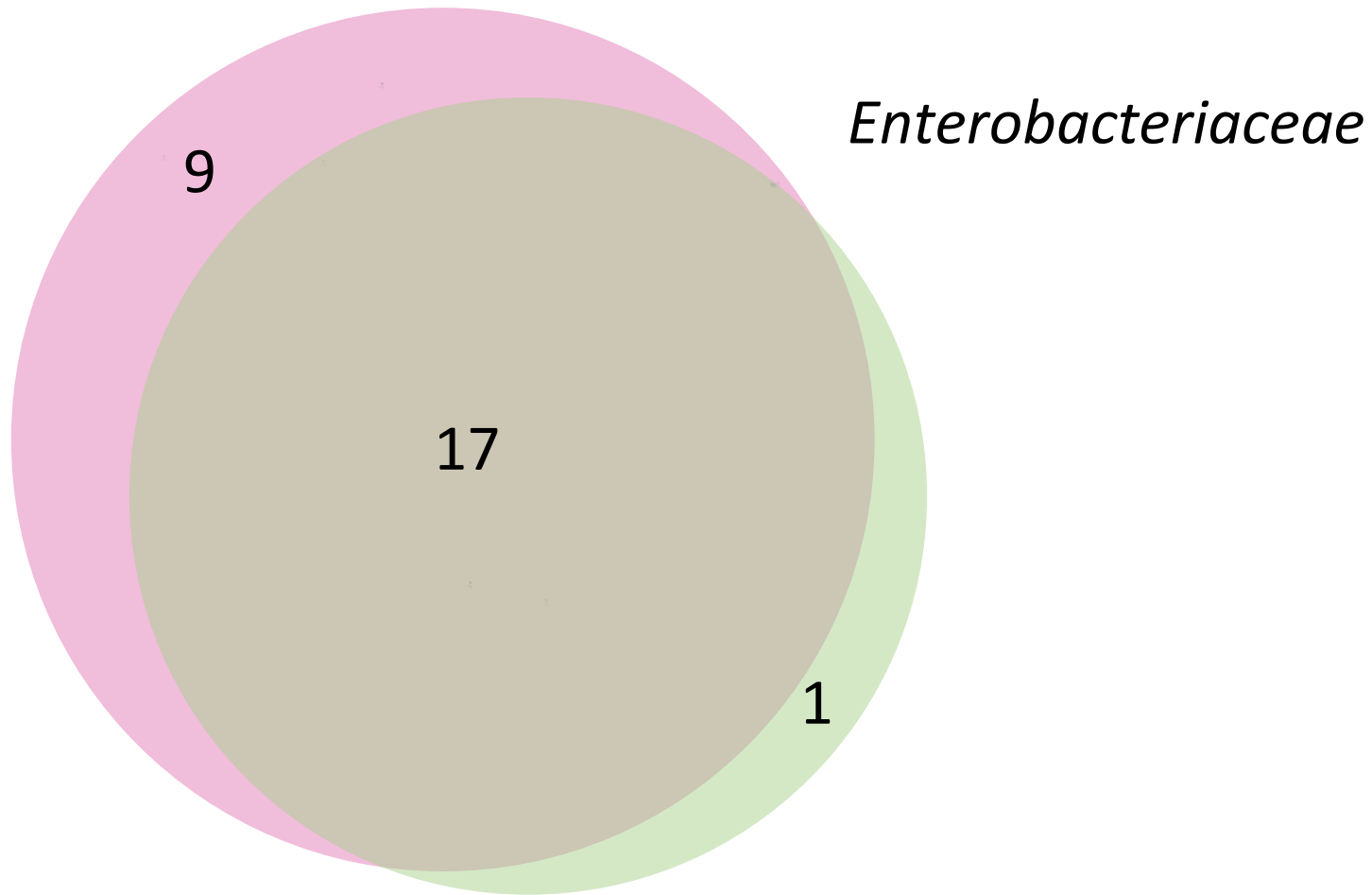

*Escherichia sp., Salmonella sp., Klebsiella sp.*

Supplement: Supplemental Information 1 — Comparison of significant domains found in genus-level and family-level analysis. Green circle represents significant domains from results for Escherichia sp., Salmonella sp. and Klebsiella sp. genomes, red circle represents domains from the Enterobacteriaceae family. [file peerj-11-15715-s001.pdf]

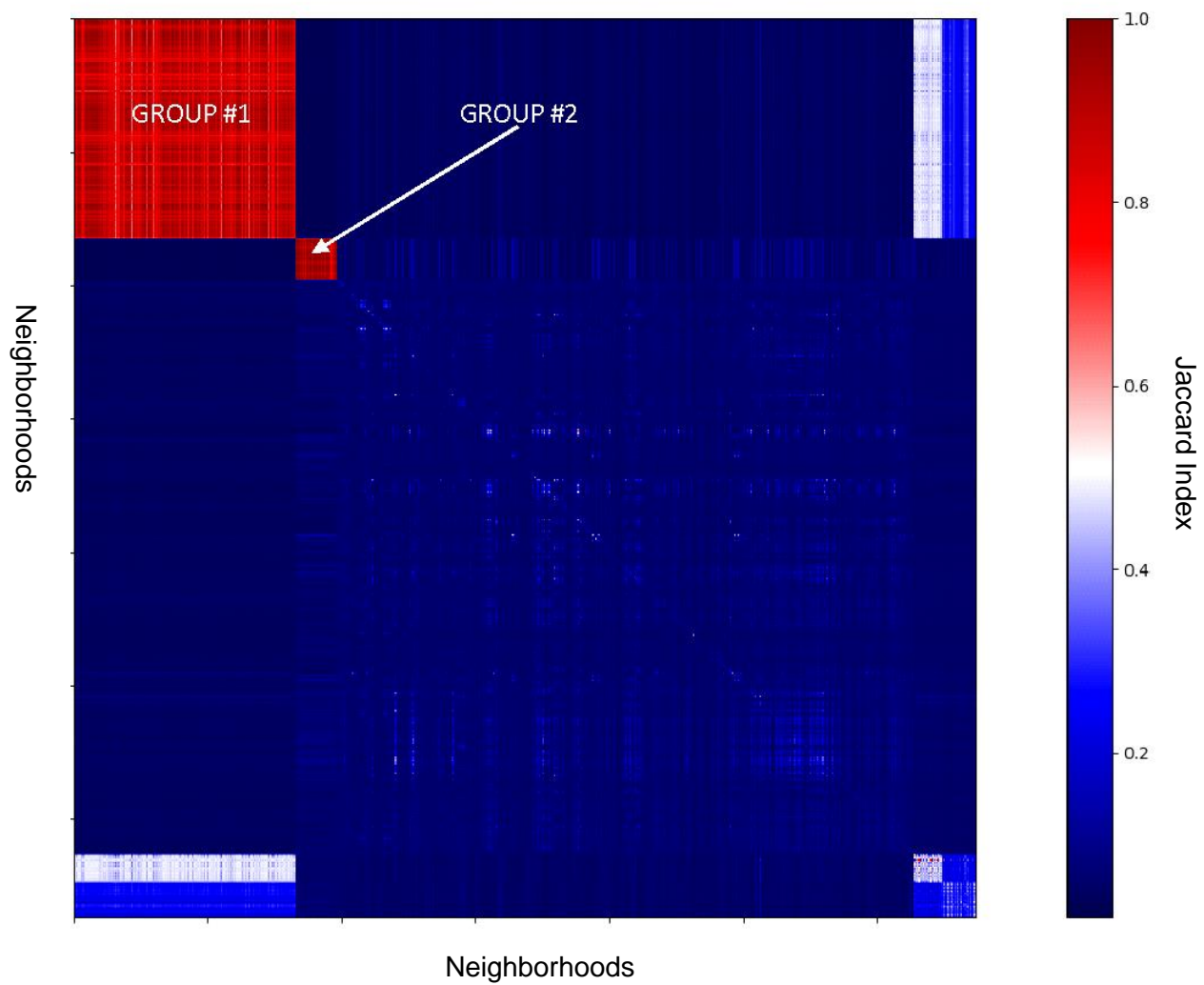

Supplement: Supplemental Information 2 — Total of 42691 neighborhoods shown. Rows and columns correspond to the SelO neighborhoods, color bar shows value of Jaccard Index, representing similarity in protein domain composition of the neighborhoods. The heatmap is clustered using single linkage clustering. [file peerj-11-15715-s002.pdf]

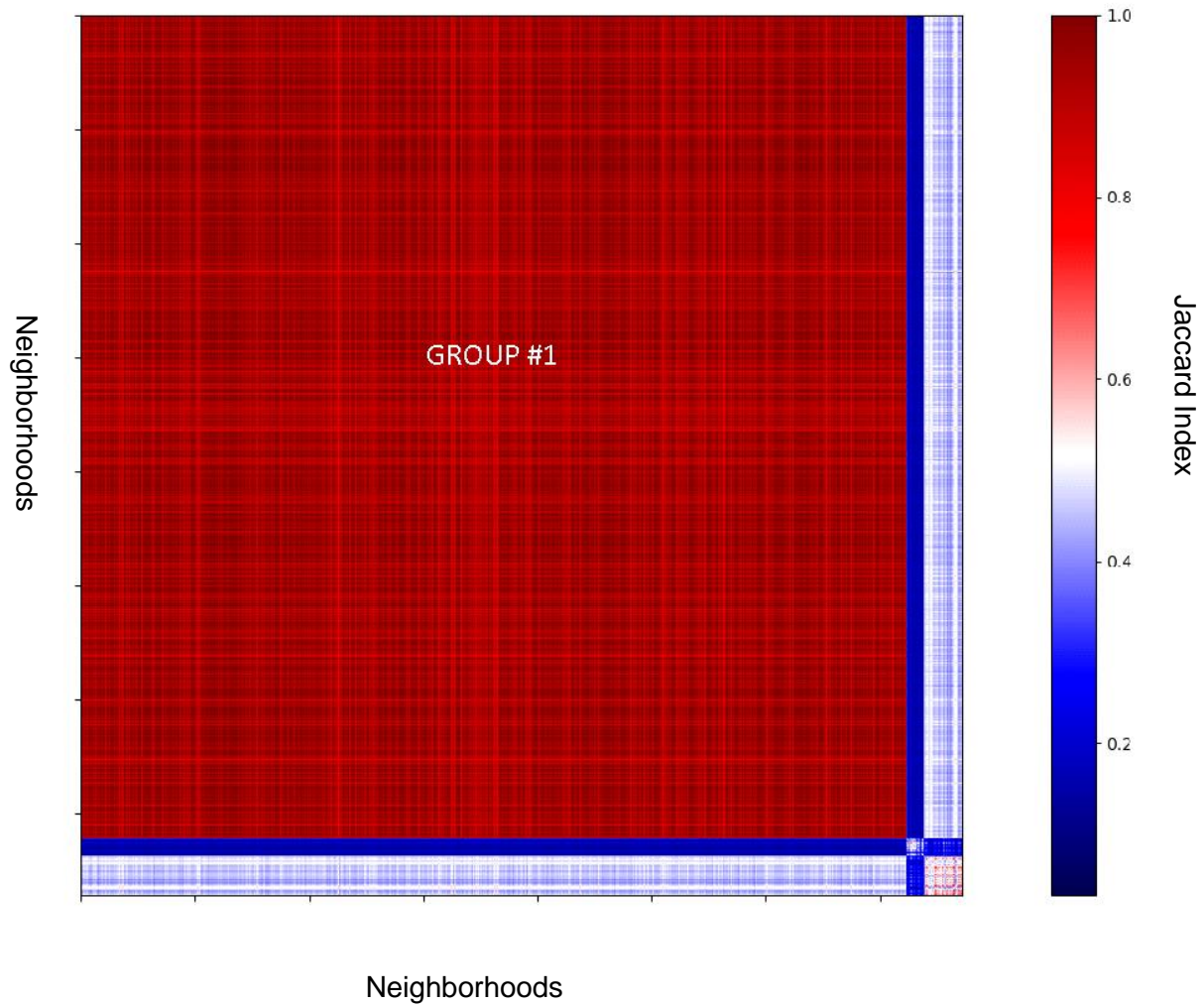

Supplement: Supplemental Information 3 — Total of 4130 neighborhoods shown. Rows and columns correspond to the SelO neighborhoods, color bar shows value of Jaccard Index, representing similarity in protein domain composition of the neighborhoods. The heatmap is clustered using single linkage clustering. [file peerj-11-15715-s003.pdf]
