# Supplementary material for: Protein family neighborhood analyzer—ProFaNA
Source: PeerJ. 2023 Jul 21;11:e15715. doi: 10.7717/peerj.15715 (PMC10364804; doi:10.7717/peerj.15715)
Supplement: Supplemental Information 4 — Related to Figure 5. Featured domains are those that occur in genomes in close proximity of SelO but very rarely elsewhere in the genomes. [file peerj-11-15715-s004.docx]

| GROUPS OF NEIGHBORHOODS | DOMINANT GENUS | FEATURED DOMAINS | NUMBER OF NEIGHBORHOODS |
| --- | --- | --- | --- |
| Group #1 | Shigella (70%) | Kinase-PPPase, hemP, PPDK_N | 448 |
| Group #2 | Enterobacteriaceae (95%) | FDX-ACB, B5, GSHPx, CoA_trans, | 383 |
| Group #3 | Salmonella (100%) | Kinase-PPPase, hemp, GSHPx, FDX-ACB, B5, PPDK_N | 1708 |
| Group #4 | Klebsiella (100%) | Kinase-PPPase, PPDK_N, hemP, | 473 |
| Group #5 | Salmonella (100%) | GSHPx, hemp, Kinase-PPPase, PPDK_N, B5, FDX-ACB | 470 |
| Group #6 | Klebsiella (100%) | Kinase-PPPase, PPDK_N, hemP | 471 |
| Group #7 | Escherichia (65%) | GSHPx, B3_4, b5, PPDK_N, | 4462 |
